# Translation and Validation of the German 12-Item Obsessive-Compulsive Inventory (OCI-12) in Clinical and Non-Clinical Samples

**DOI:** 10.32872/cpe.16165

**Published:** 2025-11-28

**Authors:** Celina L. Müller, Jakob Fink-Lamotte, Lena Jelinek, Luzie Lohse, Thomas Ehring, Michael Noll-Hussong, Götz Berberich, Andreas Wahl-Kordon, Jens Borgelt, Dean McKay, Jonathan S. Abramowitz, Amitai Abramovitch, Barbara Cludius

**Affiliations:** 1 Department of Psychology, LMU Munich, Munich, Germany; 2 Department of Psychology, Julius-Maximilians-Universität Würzburg, Würzburg, Germany; 3 Department of Clinical Psychology and Psychotherapy, University of Leipzig, Leipzig, Germany; 4 Department of Clinical Psychology, University of Potsdam, Potsdam, Germany; 5 Department of Psychiatry and Psychotherapy, University Medical Center Hamburg-Eppendorf, Hamburg, Germany; 6 Oberberg Day Clinic Munich-Westend, Munich, Germany; 7 Oberberg Hospital Windach, Windach, Germany; 8 Oberberg Hospital Schwarzwald, Hornberg, Germany; 9 Department of Psychology, Fordham University, New York, NY, USA; 10 Department of Psychology and Neuroscience, University of North Carolina at Chapel Hill, Chapel Hill, NC, USA; 11 Department of Psychology, Texas State University, San Marcos, TX, USA; 12 Department of Psychology, University of Bremen, Bremen, Germany; Philipps-University of Marburg, Marburg, Germany

**Keywords:** Obsessive-Compulsive Disorder, Obsessive-Compulsive Inventory, OCI-12, validation, diagnostic accuracy

## Abstract

**Background:**

The Obsessive-Compulsive Inventory-Revised (OCI-R) is widely used to assess symptoms of Obsessive-Compulsive Disorder (OCD). Despite its consistent factor structure, criticism on its syndromal validity has been raised. With the recent update of the commonly used diagnostic manuals, hoarding symptoms are now better captured by the diagnosis “pathological hoarding”. Furthermore, the neutralising scale suffers from relatively low psychometric properties. Consequently, a 12-item version of the scale (OCI-12), excluding hoarding and neutralising items was recently developed in English. The current study examined the psychometric properties of the German version of the OCI-12.

**Method:**

The psychometric properties of the translated German version of the OCI-12 were investigated in a German-speaking sample, consisting of 102 participants with OCD, 69 participants with an anxiety-related disorder, and 248 non-clinical controls.

**Results:**

The German version of the OCI-12 replicated the four-factor structure of the original English version, with a higher order factor of general OCD symptoms. In addition, similar to the original version, the German OCI-12 showed good internal consistency and test-retest reliability, moderate-to-good construct validity, and good-to-excellent diagnostic accuracy.

**Conclusion:**

The German version of the OCI-12 represents a syndromally valid and reliable inventory for assessing OCD symptoms. Psychometric properties are good-to-excellent and comparable to the original English version. The diagnostic sensitivity is good-to-excellent and further supports using the OCI-12 in clinical and research settings.

## Highlights

- The German version of OCI-12 possesses good-to-excellent psychometric properties.
- The OCI-12 presents a syndromally valid measure to assess OCD symptoms in German.
- Integrating the OCI-12 into routine clinical practice may improve OCD symptom assessment.

Over the last two decades, the most common questionnaire for the assessment of Obsessive-Compulsive Disorder (OCD) symptoms has been the Obsessive-Compulsive Inventory Revised (OCI-R; Foa et al., 2002) and the German version of the OCI-R (Gönner et al., 2007). Consisting of 18 items, the OCI-R assesses OCD symptoms on six dimensions (washing, checking, ordering, obsessing, neutralising, and hoarding). Yet despite its consistent factor structure demonstrated across various languages (e.g., Simos et al., 2019; Solem et al., 2010; Souza et al., 2011) and its frequent use, criticism on its syndromal validity has been raised. Particularly, although hoarding symptoms can still contribute to an OCD diagnosis when driven by obsessions (DSM-5; American Psychiatric Association, 2013, p. 241), hoarding is no longer considered a core symptom of OCD and is now classified as separate disorder (DSM-5; American Psychiatric Association, 2013; ICD-11; World Health Organization, 2019). Moreover, the subscale “neutralising” is limited to phenomena involving numeric content and suffers from low psychometric properties compared to other OCI-R subscales (Abramovitch et al., 2021; Abramowitz & Deacon, 2006; Hajcak et al., 2004).

With the aim to improve the syndromal validity of the OCI-R and adjust it to the current changes in the DSM-5 and ICD-11, Abramovitch et al. (2021) developed a 12-item English version of the OCI-R, called the OCI-12. The OCI-12 possesses good-to-excellent psychometric properties which were comparable to the original version of the OCI-R. The factor analysis evidenced that the four factors of checking, ordering, washing, and obsessing could explain the data well, with a general factor of OCD being beneficial to account for the covariances between the factors. Furthermore, the OCI-12 was able to differentiate between individuals with OCD and those with an anxiety-related disorder (ARD) or non-clinical (NCC) controls. In summary, the English version of the OCI-12 represents a valuable update of the OCI-R with a syndromally valid assessment of obsessive-compulsive symptoms and symptom dimensions.

The current study aimed to assess the psychometric properties of the German version of the OCI-12 to evaluate its utility in routine care and clinical research. We translated the OCI-12 into German and examined its factor structure, internal consistency, test-retest reliability, construct validity, diagnostic accuracy, cut-off criteria, and severity benchmarks.

## Method

### Translation Procedure

The translation process of the OCI-12 followed the translation-back-translation procedure as described by Cripps (2017) and added aspects of Beaton et al. (2000). The process is described in Supplement A.

### Study Procedure

Three groups of participants were assessed: OCD, ARD, NCC. Individuals in the clinical samples (OCD and ARD) were assessed at a single timepoint (T_1_). For test-retest reliability, the NCC sample was assessed at two timepoints (T_1_ and T_2_), with email invitations sent 14 days apart. At T_1_, all questionnaires were administered, whereas only the OCI-12 was administered at T_2_.

All questionnaires were administered online via the survey software REDCap (Harris et al., 2009). The study was approved by the ethics committee of the Faculty of Psychology and Educational Sciences of the LMU Munich (03_Mueller_b). All participants provided informed E-consent for data collection.

### Participants

For determining the target sample size, we followed the suggestions for minimum sample sizes of MacCallum et al. (1999) and Hair et al. (2019) to ensure that the factor analyses of the OCI-12 could be conducted in the total sample and the subsample of participants with OCD. We also referred to previous studies that conducted analyses with similar clinical and non-clinical samples (e.g., OCD: *n* = 44, clinical control: *n* = 44, non-clinical: *n* = 287; Aydin et al., 2014; OCD: *n* = 107, anxiety disorder: *n* = 30, depression: *n* = 40; Fink-Lamotte et al., 2021). Therefore, we predefined samples sizes of 100 participants in the OCD group, 50 participants in the ARD group, and 250 participants in the NCC group. The observed communalities (*h*^2^ = .77 for the total sample; *h*^2^ = .73 for the OCD sample) are on average larger than *h*^2^ = .60, confirming that our sample sizes are adequate for conducting factor analyses in both the OCD and total samples (according to Hair et al., 2019).

Participants were recruited between April 2022 and July 2024. General inclusion criteria were: minimum age of 18 years, no history of mania or psychotic disorders, and no acute suicidality. Further group-specific inclusion criteria are described below. In- and exclusion criteria were checked with dedicated questions and questionnaires in the survey’s start.

#### Clinical Samples

Participants with a primary OCD/ARD diagnosis within the previous six months, based on DSM-5 (American Psychiatric Association, 2013) or ICD-10 (World Health Organization, 1992) criteria, or those undergoing treatment due to OCD/ARD during this period, were recruited from collaborating clinics in Germany and another research project at the LMU Munich (https://osf.io/8gkjc). The diagnosis of OCD/ARD was given by healthcare providers (for participants recruited through cooperating clinics) or with a structured interview (Mini-DIPS, for participants recruited through another project; Margraf & Cwik, 2017).

For OCD participants, inclusion required a Yale-Brown Obsessive-Compulsive Scale (Y-BOCS; Hand & Büttner-Westphal, 1991) total score > 12 or a subscale score ≥ 8 for obsessions or compulsions as an indicator for clinically relevant OCD symptoms (see also Külz et al., 2014, 2019). The Y-BOCS was administered as self-rating (Y-BOCS-SR; Baer, 1993) for participants recruited through cooperating clinics (*n* = 64) and as interview version (Hand & Büttner-Westphal, 1991) for participants recruited through another project (*n* = 38). The final sample comprised 102 participants with OCD with Y-BOCS scores indicating moderate symptoms (*M* = 22.14, *SD* = 6.08; *M*_ObsessionSubscale_ = 11.25, *SD*_ObsessionSubscale_ = 3.39, *M*_CompulsionSubscale_ = 10.88, *SD*_CompulsionSubscale_ = 3.89).

ARD participants were excluded if they had a lifetime diagnosis of OCD. In total 69 participants with ARD fulfilled the inclusion criteria and completed the assessment. The diagnoses were as follows: 28.99% social anxiety disorder, 13.04% generalised anxiety disorder, 31.88% panic disorder, 5.8% agoraphobia, 10.14% post-traumatic stress disorder, 42.03% specific phobia^1^1As multiple anxiety disorders could be present at the same time, the percentages exceed 100%..

#### Non-Clinical Sample

Non-clinical participants were recruited via the German online panel PsyWeb (Universität Münster, 2025). Participants were screened for major psychological disorders with the simple version of the Web Screening Questionnaire (WSQ; Donker et al., 2009) and excluded if they exceeded any cut-off. Of 906 participants that gave informed consent to participate in the study and publication of their data, 383 filled out the screening questions and fulfilled the inclusion criteria. Of those, a total of 248 participants completed the first assessment, with 163 eligible for test-retest analyses after completing both assessments.

In summary, the final sample consisted of *N* = 419 participants, including *n* = 102 in the OCD group, *n* = 69 in the ARD group, and *n* = 248 in the NCC group. Sample characteristics are presented in Supplement B.

The dataset had also been used in a prior publication (Müller et al., 2025), which investigated the psychometric properties of the four-item ultra-brief Obsessive-Compulsive Inventory (OCI-4) by extracting the items from the OCI-12.

### Measures

The reliabilities of the questionnaires used in this study are provided in Supplement C. The psychometric properties of the OCI-12 will be elaborated below.

#### 12-Item Obsessive-Compulsive Inventory (OCI-12)

The OCI-12 is a 12-item self-report questionnaire measuring OCD symptoms and associated distress on a five-point Likert scale [ranging from 0 (not at all) to 4 (extremely)]. Each scale is assessed by three items, such as “I get upset if objects are not arranged properly.” for the ordering subscale, “I repeatedly check doors, windows, drawers, etc.” for the checking subscale, “I sometimes have to wash or clean myself simply because I feel contaminated.” for the washing subscale, and “I frequently get nasty thoughts and have difficulty in getting rid of them.” for the obsessing subscale. The German wording of each item and the associated subscales are displayed in Supplement D.

#### Yale-Brown Obsessive-Compulsive Scale (Y-BOCS)

The Y-BOCS was assessed as a 10-item interview (Hand & Büttner-Westphal, 1991) for participants recruited through another project and as a 10-item self-report measure (Y-BOCS-SR; Baer, 1993) for participants recruited through cooperating clinics. The Y-BOCS assesses the severity of obsessions and compulsions over the past week. Each item is rated on a five-point scale (0 to 4), with higher scores indicating higher symptom severity. While previous studies proposed that the two versions can be used interchangeably (Steketee et al., 1996), more recent investigations showed slightly higher scores in the clinician administered version (Federici et al., 2010; Hauschildt et al., 2019). In the current study, the Y-BOCS total scores did not differ significantly between the two administration modalities: Y-BOCS: *M* = 22.29, *SD* = 6.44 (completed by 38 participants recruited through another project); Y-BOCS-SR: *M* = 22.05, *SD* = 5.91 (completed by 64 participants recruited through cooperating clinics); *t*(71.61) = -0.19, *p* = .85.

#### Dimensional Obsessive–Compulsive Scale (DOCS)

The DOCS (Fink-Lamotte et al., 2021) assesses OCD symptom severity over the past month across four dimensions (i.e., contamination, responsibility for harm and mistakes, symmetry, and unacceptable/taboo thoughts). Each dimension incorporates five items rated on a five-point scale (0 to 4), with higher scores representing higher symptom severity.

#### Anxiety Sensitivity Index-3 (ASI-3)

The ASI-3 (Kemper et al., 2011) assesses anxiety sensitivity with 18 items rated on a five-point Likert scale [0 (very little) to 4 (very much)], with higher scores representing higher anxiety sensitivity.

#### Penn State Worry Questionnaire (PSWQ)

The PSWQ (Glöckner-Rist & Rist, 2014) assesses excessive and unrealistic worry using 16-items that are rated on a five-point Likert scale [1 (not at all typical of me) to 5 (very typical of me)], with higher scores indicating higher worry.

#### Patient Health Questionnaire-9 (PHQ-9)

The PHQ-9 (Löwe et al., 2002) assesses the severity of depressive symptoms throughout the past two weeks with nine items rated on a four-point scale [0 (not at all) to 3 (nearly every day)]. Higher scores indicate more severe depressive symptoms.

#### Web Screening Questionnaire (WSQ)

The adapted simple version of the WSQ (Donker et al., 2009) contains 13 questions that screen for the most common psychological disorders and acute suicidality. The original version of the WSQ has been validated and deemed as an appropriate screening tool (Donker et al., 2009; Meuldijk et al., 2017).

### Analytic Plan

The statistical analyses were performed in *R* Statistics (version 4.4.1; R Core Team, 2024), with significance set at *p* < .05. The R-code is available at OSF (Müller & Cludius, 2024S).

#### Confirmatory Factor Analysis

The factor structure of the OCI-12 was investigated by confirmatory factor analyses (CFA) using the *lavaan* package (version 0.6-19; Houben et al., 2015). Both, the four-factor structure (washing, checking, ordering, obsessing) and the four-factor structure including a higher-order factor of general OCD symptoms were investigated. We evaluated goodness of fit using the standardised root-mean-square residual (SRMR), root-mean-square error of approximation (RMSEA), the comparative fit index (CFI), and the Tucker-Lewis index (TLI). The following criteria as indicator for good model fit (Hu & Bentler, 1999; Schmitt, 2011): RMSEA ≤ 0.06; SRMR ≤ 0.08; CFI ≥ 0.95; TLI ≥ 0.95. As the multivariate normality assumption was violated (for mean and variance), we decided to use the “Maximum Likelihood with Robust Standard Errors and Mean-Variance Adjusted Test” in our CFAs. Therefore, all fit indices reported are robust fit indices. We further investigated in separate linear regression models whether each factor of the OCI-12 could predict the corresponding subscale of the DOCS.

#### Construct Validity

To examine construct validity, correlation analyses were conducted between OCI-12, Y-BOCS, and DOCS (convergent validity) and between OCI-12, ASI-3, PSWQ, and PHQ-9 (discriminant validity). Pearson’s correlation coefficients were interpreted according to Cohen (1988).

#### Reliability

For internal consistency, both Cronbach’s α and McDonald’s ω were calculated (Dunn, 2014; McDonald, 1999) and interpreted according to Hair (2009). The test-retest reliability of the OCI-12 was investigated with correlation analyses (interpreted according to Cohen, 1988), paired *t*-tests, and the two-way mixed effect intraclass correlation coefficient (ICC; interpreted according to Koo & Li, 2016) between T_1_ and T_2_ in the NCC sample.

#### Diagnostic Accuracy

We investigated group differences in the OCI-12 total and subscale scores by means of univariate (ANOVA) and multivariate analysis of variance (MANOVA). Furthermore, we conduced post-hoc Tukey Honest Significant Difference (Tukey HSD).

We investigated the diagnostic accuracy of the total score and each subscale with receiver operating characteristic (ROC) analyses. The area under the curve (AUC) was interpreted according to the criteria by Carter et al. (2016). Cut-off scores were established with the Youden Index (J; Youden, 1950).

## Results

### Confirmatory Factor Analysis

#### Confirmatory Factor Analysis in the OCD Sample

**Figure 1:**
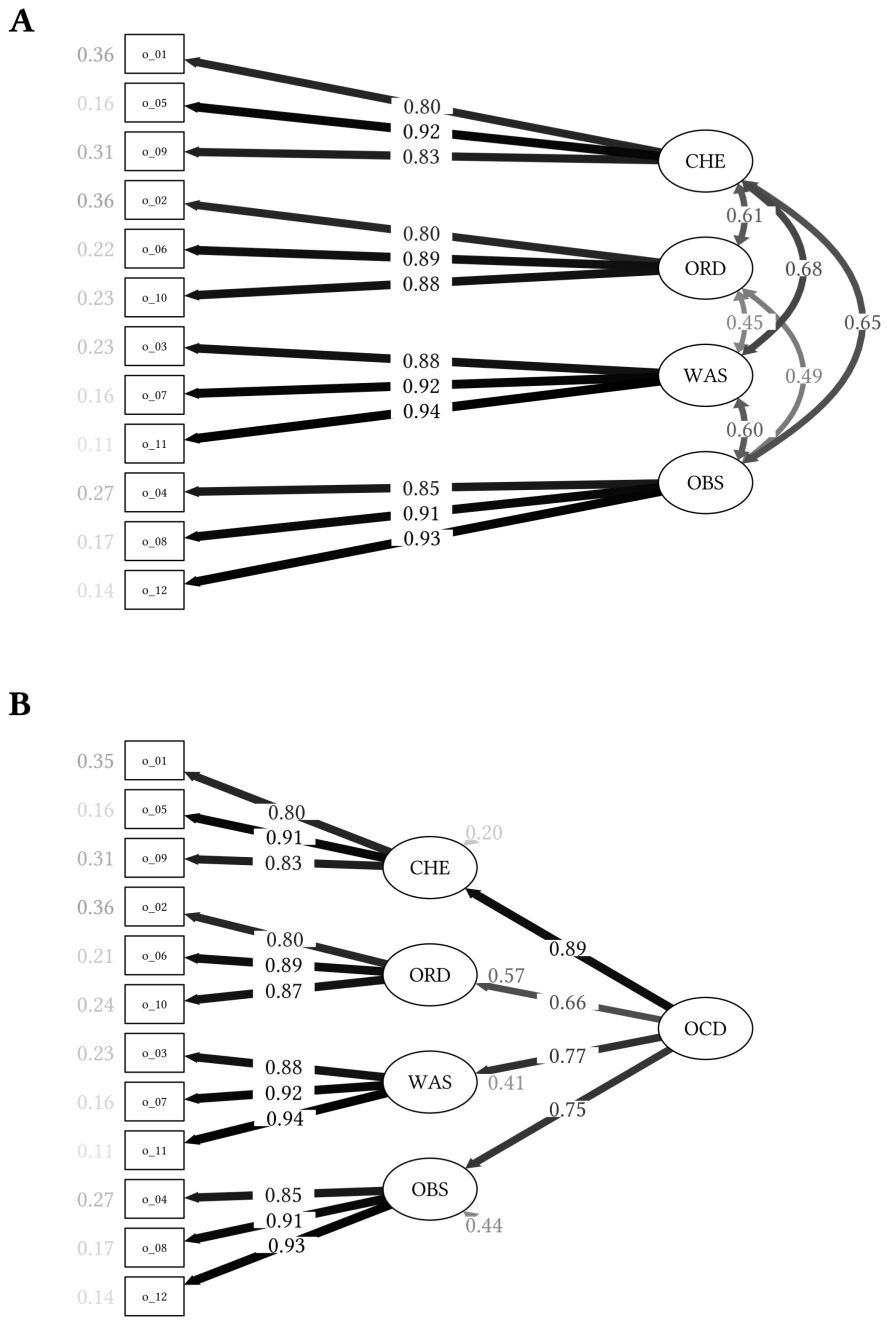
Confirmatory Factor Analyses of the OCI-12 in the OCD Sample (n = 102) *Note.* **A.** Path diagram of the structural equation model in the OCD sample depicting the four-factor model. **B.** Path diagram of the structural equation model in the OCD sample depicting the four-factor model including a higher order factor of general OCD symptoms. The factor loadings are presented between the lines, with thicker lines being indicative of higher factor loadings. Residual variances are presented next to the observed items and factors. The shading corresponds to the strength of relationships. Darker paths and values indicate stronger loadings or correlations. OCI-12 = 12-item Obsessive-Compulsive Inventory; CHE = checking subscale; ORD = ordering subscale; WAS = washing subscale; OBS = obsessing subscale; OCD = Obsessive-Compulsive Disorder.

Figure 1A displays the CFA examining the four-factor solution. The Chi-square test, χ^2^(48, *N* = 102) = 70.985, *p* = .017, rejected the hypothesis of a perfect fit. Furthermore, the TLI (0.945) did not support a good model fit while the RMSEA indicated a marginal model fit (0.081; MacCallum et al., 1996). The remaining goodness-of-fit indices supported a good fit of the four-factor model: SRMR = 0.059; CFI = 0.960.

The four-factor model including a higher-order factor of general OCD symptoms is shown in Figure 1B. Aside from the Chi-square test, χ^2^(50, *N* = 102) = 71.181, *p* = .026, and the RMSEA (0.076; reasonable fit; MacCallum et al., 1996), all fit indices support a good model fit: SRMR = 0.058; CFI = 0.963; TLI = 0.951. The first-order factors loaded weakly to strongly on the higher-order factor of general OCD symptoms. The higher-order factor accounted for a significant proportion of variance in the first-order factors checking, ordering, and washing ($R_{\mathrm{Checking}}^{2}$ = .791, $R_{\mathrm{Ordering}}^{2}$ = .286, $R_{\mathrm{Washing}}^{2}$ = .148), but not obsessing ($R_{\mathrm{Obsessing}}^{2}$ = .023).

#### Confirmatory Factor Analysis in the Total Sample

**Figure 2:**
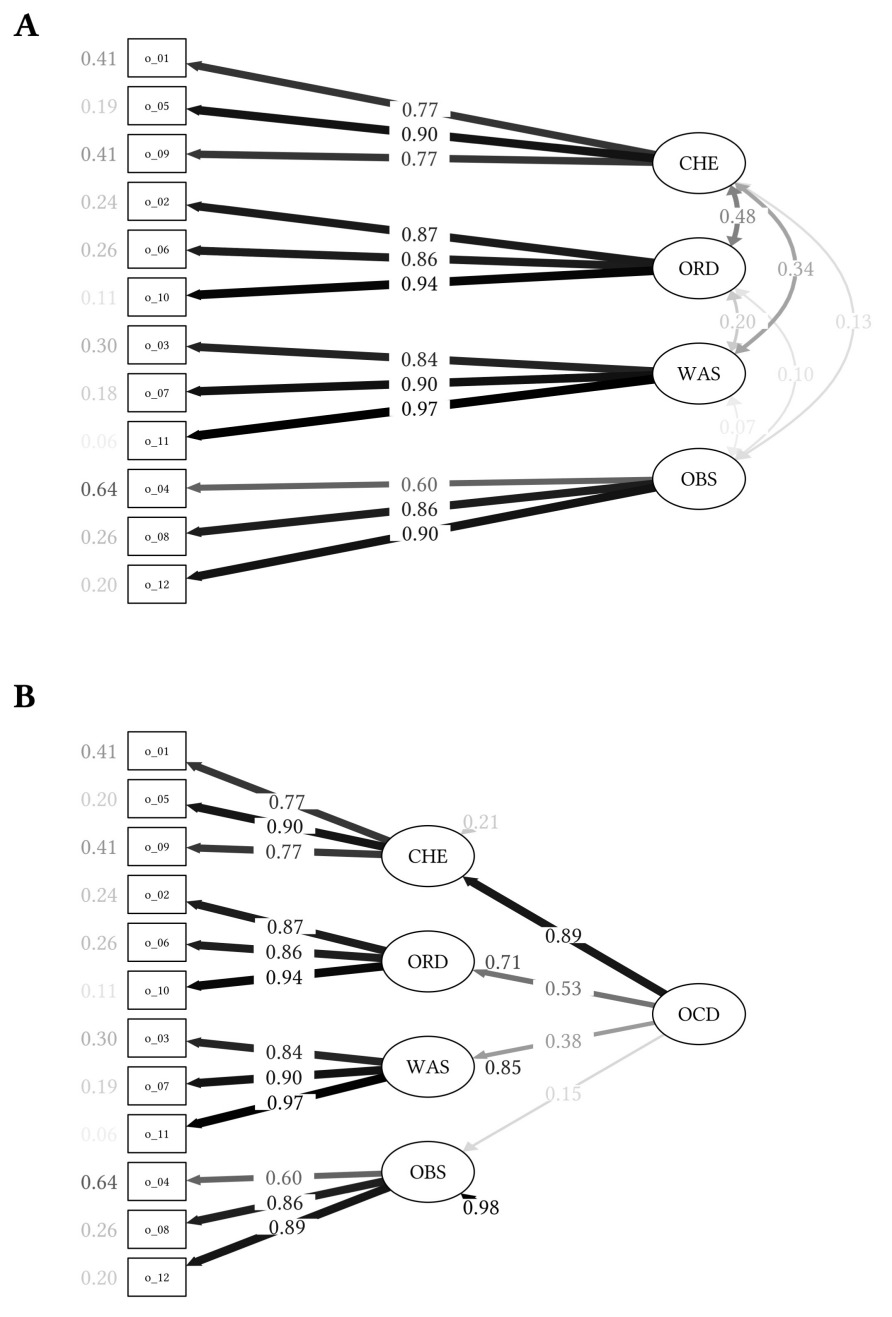
Confirmatory Factor Analyses of the OCI-12 in the Total Sample (N = 419) *Note.* **A.** Path diagram of the structural equation model in the total sample depicting the four-factor model. **B.** Path diagram of the structural equation model in the total sample depicting the four-factor model including a higher order factor of general OCD symptoms. The factor loadings are presented between the lines, with thicker lines being indicative of higher factor loadings. Residual variances are presented next to the observed items and factors. The shading corresponds to the strength of relationships. Darker paths and values indicate stronger loadings or correlations. OCI-12 = 12-item Obsessive-Compulsive Inventory; CHE = checking subscale; ORD = ordering subscale; WAS = washing subscale; OBS = obsessing subscale; OCD = Obsessive-Compulsive Disorder.

The path model of the four-factor solution in the total sample is displayed in Figure 2A. As in the OCD sample, the Chi-square test was significant, χ^2^(48, *N* = 419) = 80.558, *p* = .002) and the RMSEA showed only a reasonable fit (0.065; MacCallum et al., 1996). The remaining goodness-of-fit indices supported a good fit of the data: SRMR = 0.043; CFI = 0.979; TLI = 0.972.

Figure 2B presents the path model including the general OCD factor. Except the Chi-square test, χ^2^(50, *N* = 419) = 84.168, *p* = .002, and the RMSEA (0.065; reasonable fit; MacCallum et al., 1996), all fit indices support a good model fit: SRMR = 0.046; CFI = 0.979; TLI = 0.972. In the total sample, the first-order factors loaded strongly on the higher-order factor of general OCD symptoms. The higher-order factor accounted for a significant proportion of variance in all first-order factors ($R_{\mathrm{Checking}}^{2}$ = .796, $R_{\mathrm{Ordering}}^{2}$ = .433, $R_{\mathrm{Washing}}^{2}$ = .588, $R_{\mathrm{Obsessing}}^{2}$ = .557).

#### Correspondence of OCI-12 Subscales to DOCS Factors

The linear regression models of the four OCI-12 factors predicting each DOCS subscale are presented in Supplement E. Each DOCS subscale significantly and strongly predicted by the corresponding OCI-12 subscale (βs ranging from β = 0.44 for checking and ordering to β = 0.84 for washing).

### Reliability

#### Internal Consistency

The internal consistency for the OCI-12 subscales in the OCD group ranged from good (obsessing) to excellent (washing; see Table 1). The OCI-12 total showed good internal consistency. In the ARD subgroup, the internal consistency ranged from acceptable (washing) to good (ordering), while the OCI-12 total demonstrated good internal consistency. The NCC group showed the lowest internal consistency, ranging from not satisfactory (checking) to acceptable (obsessing), with the OCI-12 total showing acceptable internal consistency.

**Table 1:** Internal Consistency of OCI-12 Subscales per Group

OCI-12	OCD *n* = 102		ARD *n* = 69		NCC *n* = 248	
	α	ω	α	ω	α	ω
Checking	.85	.86	.78	.83	.40	.44
Ordering	.92	.92	.87	.87	.79	.70
Washing	.93	.93	.76	.76	.60	.61
Obsessing	.82	.84	.85	.85	.77	.77
**Total**	.82	.74	.84	.84	.72	.71

*Note.* OCI-12 = 12-item Obsessive-Compulsive Inventory; OCD = Obsessive-Compulsive Disorder; ARD = Anxiety-Related Disorders; NCC = Non-Clinical Controls.

#### Test-Retest Reliability

The test-retest reliability of the OCI-12 was assessed over an interval of *M* = 13.42 (*SD* = 3.64) days in the NCC group. Results are displayed in Table 2. Results of *t*-tests indicated no significant changes over the test-retest interval for OCI-12 total and all subscales except for washing, which significantly increased from T_1_ to T_2_. A strong positive correlation between T_1_ and T_2_ was shown for the OCI-12 total and a moderate (checking) to strong (ordering, washing, obsessing) positive correlation for the OCI-12 subscales. The two-way mixed effect ICC demonstrated moderate (checking) to good (ordering, washing, obsessing) reliability for the subscales and good reliability for the OCI-12 total score.

**Table 2:** Descriptives and Test-Retest Measures of the OCI-12

OCI-12	T_1_		T_2_		*t*-Test		Pearson’s Correlation		Consistency	
	*M*	*SD*	*M*	*SD*	*t*	*p*	*r*	*p*	*F*	ICC
Checking	0.80	0.99	0.72	0.96	-1.151	.251	.52	< .001	3.2	.69
Ordering	2.09	1.84	2.24	1.88	1.214	.227	.65	< .001	4.8	.79
Washing	0.33	0.75	0.47	0.96	2.506	.013	.67	< .001	4.7	.79
Obsessing	1.09	1.56	1.18	1.58	0.991	.323	.71	< .001	6.0	.83
**Total **	4.32	3.42	4.61	3.57	1.323	.188	.67	< .001	5.1	.80

*Note.* OCI-12 = 12-item Obsessive-Compulsive Inventory; ICC = two-way mixed effect intraclass correlation coefficient. These measures were obtained in the sample of Non-Clinical Controls (*n* = 163) only.

### Construct Validity

As shown in Table 3, the OCI-12 correlated moderately with the Y-BOCS in the OCD sample and strongly with the DOCS in all groups. The correlations with depressive symptoms, anxiety, and worry were moderate.

**Table 3:** Correlations Between OCI-12 and Measures of OCD Symptoms, Depression, Anxiety, and Worry per Group

Measure	OCD		ARD		NCC	
	*n*	*r*	*n*	*r*	*n*	*r*
OCD Symptoms (Convergent Validity)						
Y-BOCS_Total_	102	.45	–	–	–	*–*
DOCS_Total_	102	.74	69	.63	248	.50
Other Symptoms (Divergent Validity)						
PHQ-9	102	.48	69	.29	248	.44
ASI-3	102	.47	69	.37	248	.39
PSWQ^a^	101	.48	65	.30	237	.46

^a^Missing values in the dataset.

*Note.* OCD = Obsessive-Compulsive Disorder; ARD = Anxiety-Related Disorders; NCC = Non-Clinical Controls; OCI-12 = 12-item Obsessive-Compulsive Inventory; Y-BOCS = Yale-Brown Obsessive-Compulsive Inventory; DOCS = Dimensional Obsessive-Compulsive Scale; PHQ-9 = Patient-Health Questionnaire-9; ASI-3 = Anxiety-Sensitivity Index-3; PSWQ = Penn-State Worry Questionnaire.

### Diagnostic Accuracy

#### Group Differences

Descriptives and group differences on the OCI-12 are presented in Table 4. The total scores of the OCI-12 significantly differed between groups, *F*(2, 416) = 373.1, *p* < .001, as indicated by a main effect of group in the ANOVA. Participants with OCD had significantly higher OCI-12 scores than participants with ARD and NCC, which showed significantly higher OCI-12 scores than the NCC group (all *p*’s < .001 in Tukey’s HSD tests).

**Table 4:** Descriptives and Group Differences of the OCI-12

OCI-12	OCD *n* = 102				ARD *n* = 69				NCC *n* = 248			
	*M*	*SD*	*Mdn*	*IQR*	*M*	*SD*	*Mdn*	*IQR*	*M*	*SD*	*Mdn*	*IQR*
Checking	5.26	3.32	5	6	2.06	2.32	1	3	0.79	0.95	1	1
Ordering	4.90	3.52	4	5.75	3.78	3.14	3	5	1.96	1.74	2	2
Washing	5.83	4.41	6	8.75	1.59	2.16	1	3	0.34	0.85	0	0
Obsessing	7.22	3.06	8	4	3.57	2.74	3	5	1.02	1.43	1	2
**Total**	23.22	9.19	23	12	11.00	7.34	10	10	4.10	3.23	3	4

*Note.* The values for each subscale can range from 0 – 12. The possible range for the total score is 0 – 48. OCI-12 = 12-item Obsessive-Compulsive Inventory; OCD = Obsessive-Compulsive Disorder; ARD = Anxiety-Related Disorders; NCC = Non-Clinical Controls; *Mdn* = Median; IQR = Interquartile Range.

When considering the OCI-12 subscales, the MANOVA showed a significant main effect of group across the subscales, Pillai’s Trace = 0.733, *F*(8, 828) = 59.837, *p* < .001. As for the OCI-12 total score, separate ANOVAs with post-hoc Tukey HSD tests revealed that participants with OCD had significantly higher scores on each subscale than participants with ARD, which showed significantly higher scores than the NCC group (all *p*’s < .001).

#### Diagnostic Accuracy

**Figure 3:**
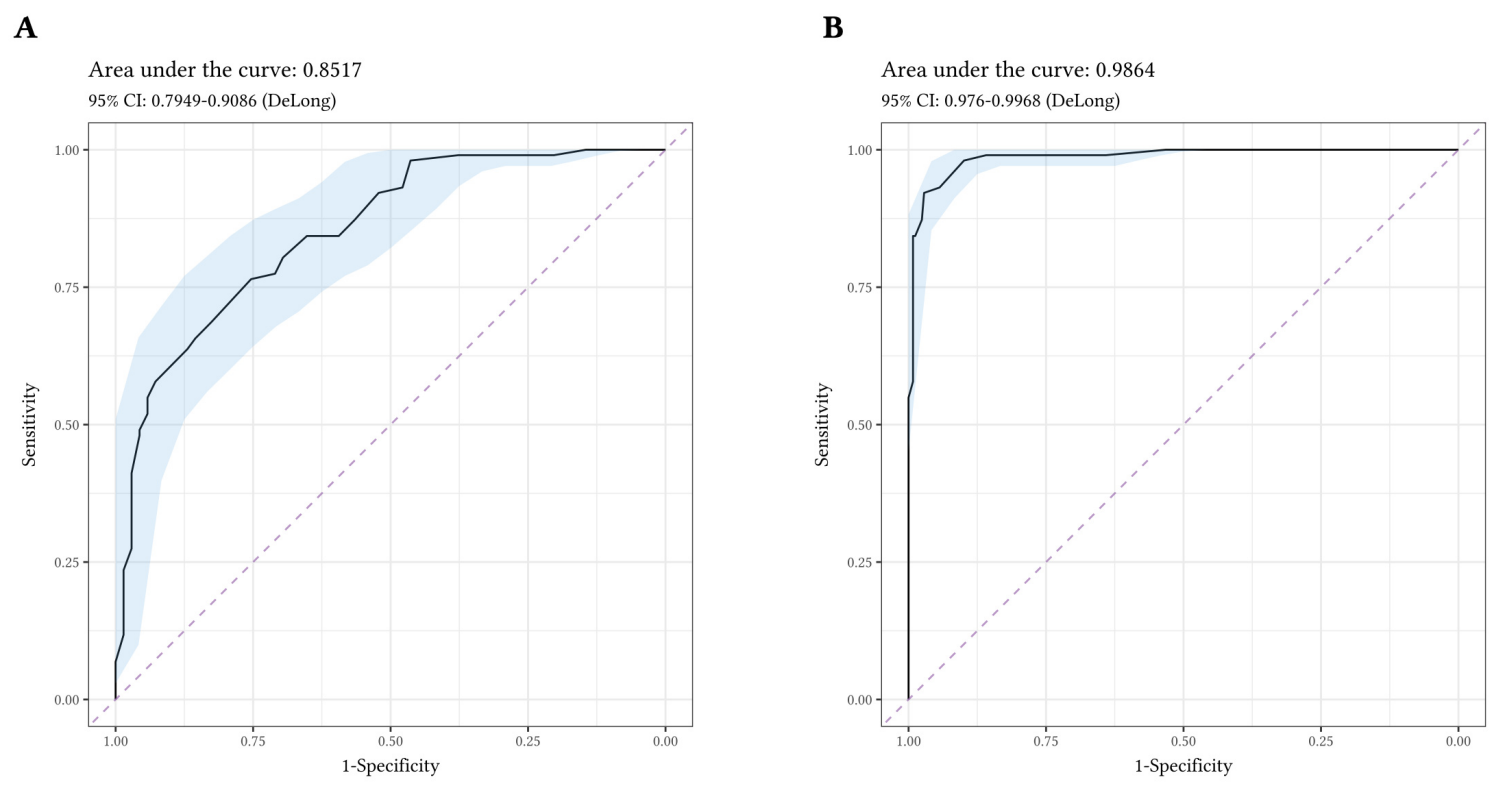
Receiver Operating Characteristic Curves for the OCI-12 Total *Note.* **A.** Receiver operating characteristic (ROC) curve discriminating participants with Obsessive-Compulsive Disorder (*n* = 102) from participants with Anxiety-Related Disorders (*n* = 69). **B.** Receiver operating characteristic (ROC) curve discriminating participants with Obsessive-Compulsive Disorder (*n* = 102) from Non-Clinical Controls (*n* = 248).

**Figure 4:**
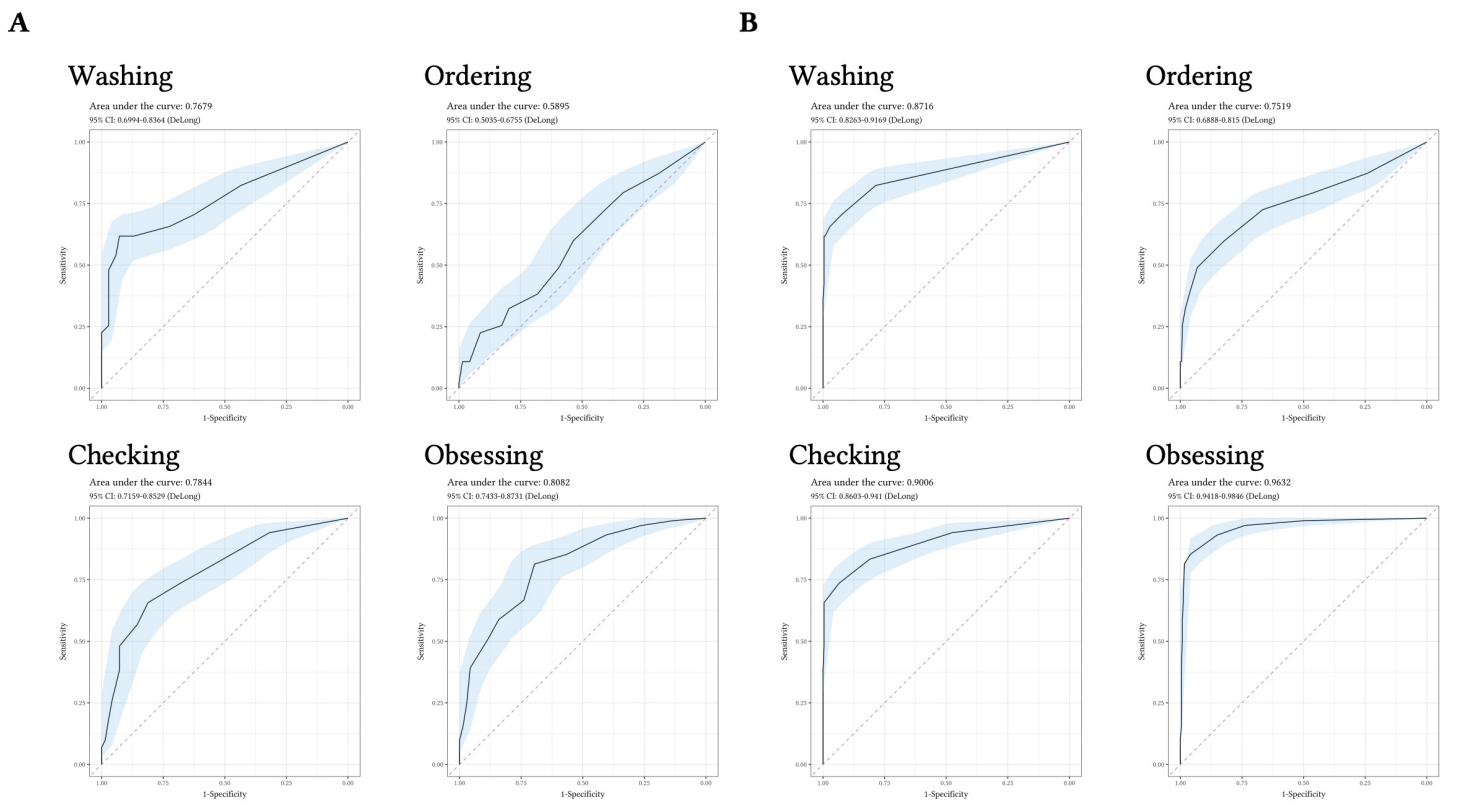
Receiver Operating Characteristic Curves for the OCI-12 Subscales *Note.* **A.** Receiver operating characteristic (ROC) curves for the OCI-12 subscales discriminating participants with Obsessive-Compulsive Disorder (*n* = 102) from participants with Anxiety-Related Disorders (*n* = 69). **B.** Receiver operating characteristic (ROC) curves for the OCI-12 subscales discriminating participants with Obsessive-Compulsive Disorder (*n* = 102) from Non-Clinical Controls (*n* = 248).

The diagnostic accuracy of the OCI-12 to discriminate participants with OCD from participants with ARD was good (AUC = .85, 95% CI [.795, .909]; see Figure 3A). The diagnostic accuracy for each subscale ranged from AUC = .59 (ordering) to AUC = .81 (obsessing; see Figure 4A).

Considering the diagnostic accuracy of the OCI-12 for distinguishing individuals with OCD from NCC’s, the OCI-12 total score evidenced excellent accuracy (AUC = .99, 95% CI [.976, .997]; see Figure 3B). The diagnostic accuracy of the subscales ranged from AUC = .75 (ordering) to AUC = .96 (obsessing). Overall, the OCI-12 total score evidenced the best diagnostic accuracy for discriminating OCD participants from both, ARD and NCC (see Figure 4B).

#### Optimal Cut-Off

Table 5 summarises the Youden Indices, sensitivities, and specificities of the OCI-12 total and each subscale for discriminating participants with OCD from ARD and NCC participants. An OCI-12 total score ≥ 17 was considered optimal to discriminate participants with OCD from participants with ARD. When discriminating participants with OCD from NCC’s, a score of ≥ 11 was considered optimal. Out of the subscales, the washing subscale could best discriminate OCD from ARD participants, while the obsessing subscale best discriminated OCD from NCC participants. The ordering subscale was suited worst to discriminate participants with OCD from ARD and NCC participants.

**Table 5:** Optimal Cut-Offs for OCI-12 Subscales

OCI-12 Subscale	OCD vs. ARD				OCD vs. NCC			
	Cut-Off	J	Sensitivity	Specificity	Cut-Off	J	Sensitivity	Specificity
Checking	4	.47	65.69%	81.16%	3	.67	73.53%	93.55%
Ordering	9	.14	22.55%	91.30%	5	.42	49.02%	93.15%
Washing	5	.55	61.76%	92.75%	2	.63	70.59%	92.34%
Obsessing	5	.51	81.37%	69.57%	4	.81	85.29%	95.97%
**Total**	17	.52	76.47%	75.36%	11	.89	92.16%	97.18%

*Note.* OCI-12 = 12-Item Obsessive-Compulsive Inventory; OCD = Obsessive-Compulsive Disorder; ARD = Anxiety-Related Disorders; NCC = Non-Clinical Controls; J = Youden Index; Sensitivity = correct classification of OCD participants; Specificity = correct classification of non-OCD participants.

#### Severity Benchmarks

OCI-12 severity benchmarks were investigated in severity-groups based on Y-BOCS cut-offs (Storch et al., 2015; see Supplement F for OCI-12 descriptives per severity group). The OCI-12 total score fairly discriminated mild from moderate cases (AUC = .73, 95% CI [.557, .900]), but only poorly distinguished moderate from moderate-severe cases (AUC = .69, 95% CI [.576, .807]) and moderate-to-severe from severe cases (AUC = .52, 95% CI [.059, .983]).

 Given that the severe group included only three individuals, moderate-to-severe and severe cases were combined, but discrimination from moderate cases remained poor (AUC = .69; 95% CI [.577, .801]). An optimal cut-off ≥ 12 was suggested for mild vs. moderate cases (J = .38), and a cut-off ≥ 24 for moderate vs. moderate-severe cases (J = .38). Due to small sample sizes and limited discrimination, further research with a larger sample is needed to establish OCI-12 severity benchmarks.

## Discussion

To utilise the OCI-12 in German-speaking populations, we translated the original English version (Abramovitch et al., 2021) into German and investigated its psychometric properties. We replicated the original four-factor structure with a higher-order factor of general OCD symptoms. Furthermore, our results on the reliability, validity, and diagnostic accuracy of the OCI-12 are good-to-excellent and comparable to the original English version.

More specifically, the four-factor model (washing, checking, ordering, and obsessing) including the higher-order factor of general OCD symptoms showed a good fit to the data according to the CFAs. The higher-order factor also explained significant variance in the OCI-12 subscales. Of note, the chi-square test and the RMSEA did not support a good model fit. However, both indices are criticised for being sensitive to the sample size (Bollen, 2014; Hu & Bentler, 1999) and the degrees of freedom (Kenny et al., 2015), respectively. As most of the approximate fit indices (i.e., SRMR, CFA, TLI) supported a good fit of the data, we conclude that the four-factor structure with a higher-order factor of general OCD symptoms is also evident in the German-speaking sample. Moreover, each of the OCI-12 subscale significantly and most strongly predicted the corresponding subscale of the well-established DOCS, providing further evidence for the four factors.

The OCI-12 total score’s internal consistency and test-retest reliability was good. In terms of construct validity, the current correlation analyses showed only a moderate correlation between OCI-12 scores and the Y-BOCS. This relatively low correlation has also been shown in previous studies (see Abramovitch et al., 2021; Aspvall et al., 2020) and may be related to idiographic nature of the Y-BOCS in measuring OCD symptom severity of individually assessed obsessions and compulsions as compared to the nomothetic approach of the OCI-12. Moreover, the format of administration seems to contribute to the relatively low correlation, hinting towards the *common method bias* (Podsakoff et al., 2003). Indeed, an exploratory correlation analysis showed that the correlation between the two self-reports, OCI-12 and Y-BOCS-SR, was higher (*r* = .56, *p* < .001) than the correlation between the OCI-12 and the Y-BOCS interview (*r* = .28, *p* = .090). However, in support of convergent validity, the correlation between the OCI-12 and the DOCS is strong. Therefore, we consider the comparably weak correlation with the Y-BOCS rather as a methodological/conceptual artefact. Correlations between the OCI-12 and measures of depression, anxiety, and worry have been moderate, highlighting that the OCI-12 possesses discriminant validity but is not completely independent of these symptom measures. Given that the clinical samples present with comorbid diagnoses (e.g., depression), these results are, however, not surprising.

When comparing the OCI-12 scores between the three groups, the group of participants with OCD showed significantly higher scores than both, participants with ARD and NCC. The OCI-12 can discriminate well between participants with OCD and ARD when a cut-off of ≥ 17 is considered and can discriminate excellently between participants with OCD and NCC’s when a cut-off of ≥ 11 is used. Of note, the OCI-12 should not be considered as isolated diagnostic tool (i.e., the cut-off criteria should not replace a diagnostic interview). Analyses of the severity benchmarks showed that the OCI-12 could fairly discriminate mild from moderate cases, but only poorly discriminate between cases of mild or severe symptom severity. However, due to the small sample sizes within the severity groups, future research is needed to establish conclusive benchmarks.

### Limitations

This study has some limitations. Although participants were recruited within cooperating clinics, structured diagnostic interviews were not always possible, risking less precise diagnoses, particularly for comorbid disorders. Likewise, the absence of structured interviews for the NCC population may have allowed the inclusion of participants with undiagnosed psychological disorders, not captured by dedicated questions or the WSQ.

Furthermore, 63% of participants with OCD filled out the Y-BOCS as self-rating, whereas 37% completed the Y-BOCS interview. While both formats show strong correlations and good reliability and may be used interchangeably (Baer et al., 1993; Rosenfeld et al., 1992; Steketee et al., 1996), the weak correlation between the interview version and the OCI-12 hints towards a *common method bias,* which should be taken into consideration when interpreting the convergent validity. As the correlation between the OCI-12 and the Y-BOCS is affected by the assessment modality, it may be worthwhile in future studies to investigate the correlation between the OCI-12 and other interview-based measures.

Additionally, participants in the clinical samples presented with comorbid disorders. Although this increases the ecological validity of the current validation, comorbid information is not integrated into the psychometric analyses. Therefore, comorbid symptoms may attenuate some of the reported measures (e.g., internal validity, discriminant validity).

Lastly, the sample size of the OCD group should be increased in future studies. While the average item communalities (*h*^2^ = .73) indicate that a sample size of *n* = 100 is adequate for conducting the CFA according to rules of thumb (Hair et al., 2019), a larger OCD sample may enhance the robustness of findings. The total sample size (*N* = 419), however, is adequate for conducting the CFA on the OCI-12 which supports the four-factor structure, including the higher-order factor representing general OCD symptoms. A valuable next step would be recruiting a large, representative sample, which would allow the development of norms for the OCI-12.

### Conclusion

The German version of the OCI-12 presents a syndromally valid self-report measure to assess OCD symptoms which can be used in research and clinical settings. The original four-factor structure with a higher-order factor of general OCD symptoms could be replicated and the OCI-12 possesses good-to-excellent psychometric properties in terms of internal and test-retest reliability and construct validity. Furthermore, the OCI-12 possesses good-to excellent-diagnostic accuracy for its established clinical cut-off values. To enable a wide use of the OCI-12, the German versions of this questionnaire, including the item numbering and scoring guidelines, can be found in Supplement G and in Müller and Cludius, 2024S. As a next step, conducting a study with larger sample sizes would be valuable to establish norms, enabling an even more meaningful and precise interpretation of scores on the OCI-12.

## Supplementary Materials

The Supplementary Materials contain the following items:

- *Research data, codebook, and code* (Müller & Cludius, 2024S)
- *Additional information* (Müller et al., 2025S):
  - Supplement A: Translation Procedure
  - Supplement B: Sample Characteristics
  - Supplement C: Internal Consistencies of Questionnaires
  - Supplement D: Formulation of OCI-12 Items
  - Supplement E: OCI-12 Subscales Predicting DOCS Factors
  - Supplement F: Severity Benchmarks
  - Supplement G: 12-Item Obsessive-Compulsive Inventory (OCI-12)

MüllerC. L.
CludiusB.
 (2024S). German Version OCI-12
[Reseach data, codebook, and code]. PsychOpen. https://osf.io/4m9x6/


MüllerC. L.
Fink-LamotteJ.
JelinekL.
LohseL.
EhringT.
Noll-HussongM.
BerberichG.
Wahl-KordonA.
BorgeltJ.
McKayD.
AbramowitzJ. S.
AbramovitchA.
CludiusB.
 (2025S). Supplementary materials to "Translation and validation of the German 12-item Obsessive-Compulsive Inventory (OCI-12) in clinical and non-clinical samples"
[Additional information]. PsychOpen. 10.23668/psycharchives.21291
PMC1292319841725700

## Data Availability

The research data, codebook, and code are provided at the Open Science Framework (Müller & Cludius, 2024S).

## Author Note

The present manuscript details the psychometric properties of the German version of the OCI-12. In a separate publication (Müller et al., 2025), we conducted a first investigation of the German four-item ultra-brief Obsessive–Compulsive Inventory (OCI-4), which was derived from the dataset used in this study by selecting four items from the OCI-12.

## Social Media Accounts

*Celina Müller:* LinkedIn, Bluesky

## Preregistration

Analyses were not preregistered.

## References

[sp1_r1] Müller C. L. Cludius B. (2024 S). German Version OCI-12 [Reseach data, codebook, and code]. PsychOpen. https://osf.io/4m9x6/

[sp1_r2] Müller C. L. Fink-Lamotte J. Jelinek L. Lohse L. Ehring T. Noll-Hussong M. Berberich G. Wahl-Kordon A. Borgelt J. McKay D. Abramowitz J. S. Abramovitch A. Cludius B. (2025 S). Supplementary materials to "Translation and validation of the German 12-item Obsessive-Compulsive Inventory (OCI-12) in clinical and non-clinical samples" [Additional information]. PsychOpen. 10.23668/psycharchives.21291 PMC12923198 41725700

